# Blood lipid profile and body composition in a pediatric population with different levels of physical activity

**DOI:** 10.1186/s12944-018-0817-2

**Published:** 2018-07-25

**Authors:** Joanna Baran, Aneta Weres, Ewelina Czenczek-Lewandowska, Justyna Wyszyńska, Edyta Łuszczki, Katarzyna Dereń, Grzegorz Sobek, Paweł Więch

**Affiliations:** 1 Institute of Physiotherapy, Medical Faculty, University of Rzeszów, Rzeszów, Poland; 2 Institute of Nursing and Health Sciences, Medical Faculty, University of Rzeszów, Rzeszów, Poland; 3 Centre for Innovative Research in Medical and Natural Sciences’, Medical Faculty, University of Rzeszów, Kopisto 2a, 35-959 Rzeszów, Poland

**Keywords:** Accelerometry, Adolescent, Fat content, Lipids, Children

## Abstract

**Background:**

Associations between physical activity and lipid/lipoprotein levels and body composition among pediatric populations are not completely consistent in the literature. Accordingly, the aim of this study was to analyze lipid and lipoprotein profiles and body composition among children and adolescents differentiated according to their level of physical activity.

**Methods:**

The study sample consisted of 69 participants that ranged from 6 to 17 years of age. Objective measures of physical activity were obtained over 7 days with accelerometers. Total cholesterol (TC), low density lipoprotein (LDL), high density lipoprotein (HDL) and triglycerides (TG) were measured from a fasting blood sample. High-risk values for these lipids/lipoproteins were determined using age- and sex-specific thresholds. Body composition estimates were obtained using a foot-to-foot bioelectrical impedance analysis.

**Results:**

Almost half (47.8%) of the participants did not meet daily moderate-to-vigorous intensity physical activity (MVPA) recommendations. This group had lower free fat mass (by 5.51%), muscle mass content (by 5.17%), and a higher risk for abnormal HDL level (OR = 4.19) and excessive body fat content (OR = 3.05).

**Conclusions:**

Participants who met daily recommended MVPA were found to have more favorable HDL and body fat levels compared to those who did not meet these recommendations.

## Background

The prevalence of childhood obesity is a major public health issue and has been increasing dramatically over the last decades. Globally, it was estimated that in 1990, 4.2% of children had excessive body mass and this proportion increased to 6.7% in 2010. By the year 2020, the prevalence of overweight in the pediatric population is projected to be 9.1% which represents about 60 million children and adolescents [1]. Epidemiological research has demonstrated that obesity and low level of physical activity contribute significantly to the prevalence of cardiovascular disease. In addition, it has been found that the conditions of overweight and obesity tend to remain stable from birth through childhood and adolescence to adulthood [2, 3]. Physical activity patterns also tend to remain the same from childhood to adolescence and adulthood [4].

The World Health Organization (WHO) recommends that children aged 5–17 years should accumulate at least 60 min of moderate-to-vigorous intensity physical activity (MVPA) each day to achieve optimal health benefits [5]. Despite these recommendations, a trend of physical inactivity is increasing rapidly in most societies around the world. According to Hallal et al., 80% of children and adolescents in 105 countries fail to meet levels of physical activity recommended by WHO [6]. Low physical activity is associated with an increased incidence of obesity, cardiovascular disease, and a widening variety of other chronic diseases including hypertension, diabetes mellitus, cancer, bone and joint diseases, and depression [7–9].

Some studies have found significant relationships between levels of physical activity and serum lipid and lipoprotein levels [10–13], and body composition [14–16]. However, these associations are not completely consistent in the literature [17]. There are also studies that report a lack of association between physical activity and lipid profile [18, 19] or fat content [20]. Moreover, most of the reports refer to adults and non-objective measurements for assessment of physical activity level were used. Therefore, the purpose of the present study was to analyze the lipid profile and body composition in children and adolescents differentiated according to physical activity levels measured objectively. To accomplish this, we evaluated lipid profile, body fat percentage, free fat mass, muscle mass content, and total body water in a group of subjects meeting and not meeting daily levels of recommended MVPA.

## Methods

The study was approved by the local Bioethics Committee (No. 2/10/2015). The study was conducted from March to June 2016, after obtaining written consent from the participating children’s parents, and the children themselves. Anthropometric measurements and blood tests were carried out in the Centre for Innovative Research in Medical and Natural Sciences (Rzeszów, Poland). All measurements were taken between 7:00 AM and 10:00 AM by experienced researchers.

### Participants

The study included healthy children and adolescents aged 6 to 17 years who were enrolled in randomly selected schools in southeastern Poland during the 2015/2016 school year. The invitation to participate in the study was sent to 500 parents of children attending the schools. All participants and parents were fully informed in writing and verbally about the nature of the study. The consent of 212 parents was obtained for child participation in this study. Of those respondents, 143 were excluded from the study for the following reasons: an age of less than 6 or greater than 17 years (*n* = 12), a functional state that does not allow for self-maintenance of a standing position (*n* = 2), strong anxiety of blood test (*n* = 5), a failure to return or complete the survey (*n* = 57), removal of the accelerometer at any time of the study period/ the device suffered mechanical error/ or operator error (incorrect epoch length, anthropometrics, and/or participant identification) (*n* = 38), and refusal to participate in study (*n* = 29). Ultimately, the study group consisted of 69 students (30 girls and 39 boys) aged 6–17 years (mean age was 11.6 ± 2.74 years).

### Anthropometric measurements and body mass index

Body height was measured to the nearest 0.1 cm using a portable stadiometer Seca 213. The measurement was performed under standard conditions, in an upright position, barefoot. Body mass was assessed with an accuracy of 0.1 kg using a body composition analyzer (BC-420, Tanita).

Body mass index (BMI) was calculated as weight (kg)/height (m)^2^. Based on BMI values, the BMI percentile of individual participants were calculated. BMI percentile charts specific for age, sex, and body height were used. Percentile charts which were developed within the framework of the Polish project entitled “Developing standards of blood pressure in children and adolescents in Poland, OLAF” were used [21]. Based on the BMI percentile values, underweight (<5th percentile), healthy weight (between 5th and 85th percentile), overweight (BMI ≥85th percentile and < 95th percentile), or obesity (≥95th percentile) were determined. The definitions of underweight, healthy weight, overweight, and obesity were based on the recommendations of the Centers for Disease Control and Prevention [22].

### Bioelectrical impedance

Body composition estimates were obtained using a Tanita device (BC-420) which uses foot-to-foot bioelectrical impedance analysis (BIA). All measurements were performed in the early morning according to the guidelines of the manufacturer, with a frequency of 50 kHz. The bioelectrical impedance analysis method consists of measuring the impedance (electrical resistance, which consists of resistance and reactance) of tissue through which electrical current of low intensity is passed (< 1 mA) [23]. Measurements were taken after an overnight fast (for at least 8 h) because food or beverage consumption may decrease impedance by 4–15 Ω over a 2–4 h period after meals, representing an error smaller than 3% [24]. The foot-to-foot method of BIA is a reliable and accurate tool for the measurement of body composition in pediatric population [25]. The body fat (%), skeletal muscle content (%), free fat mass (%), total body water (%) has been analyzed. On the basis of one of the largest studies on adipose tissue and risk factors, it was found that excessive body fat levels (≥25% in boys and ≥ 30% in girls) were associated with greater health risk of cardiovascular diseases, diabetes, and other metabolic diseases [26]. Therefore, two groups of subjects were distinguished: (i) healthy body fat percentage: < 25 and < 30% of body fat in boys and girls, respectively; (ii) excessive body fat percentage: ≥ 25 and ≥ 30% of body fat in boys and girls, respectively.

### Physical activity assessment

Physical activity of the children was assessed using a triaxial accelerometer wGT3X-BT monitor (ActiGraph, LLC, Pensacola, Florida, USA). It is a lightweight accelerometer that is one of the most commonly used devices for assessing physical activity [27]. The ActiGraph was validated and tested for reliability before use [28, 29]. The accelerometer was placed on the waist using an elastic belt securely above the right hip bone for measuring the amount and frequency of participant movement. The accelerometers were initialized at a sampling rate of 30 Hz to record activities for free-living conditions. Subjects were instructed to wear the accelerometer 24 h a day for seven consecutive days, including the hours they were sleeping. They were advised to remove the accelerometers only during water-based activities such as bathing or immersing the body in water. Parents were also asked to ensure that the accelerometers were worn at all times, however, participants were permitted to remove the accelerometers if they felt uncomfortable wearing the device.

Data was collected in 60s epochs. Non-wear time was defined as 60 min of consecutive zeros allowing for 2 min of non-zero interruptions [30]. A wear time of ≥500 min/day was used as the criterion for a valid day, and ≥ 4 days were used as the criteria for a valid 7-day period of accumulated data. Data was accessible by using the ActiLife Pro 6 software (ActiGraph LLC, Pensacola, Florida, USA). We characterized average intensity of physical activity using average counts per minute (CPM). Using Evenson’s cut-off points, we defined various intensities of physical activity including sedentary (0–100 CPM), light (101–2295 CPM), moderate (2296–4011 CPM), vigorous (> 4012 CPM), and MVPA (> 2296 CPM) [31]. Participants with at least 60 min of MVPA per day were considered to have met the physical activity guidelines (active), and those with daily MVPA < 60 min were considered to have not met physical activity guidelines (inactive) [5].

### Blood lipids

Blood lipids were analyzed by a pediatric nurse who received a fasting blood sample from each enrolled participant by finger prick. Participants were advised to fast for 10–12 h before the test. Blood was analyzed immediately using the Cholestech LDX Analyzer (Cholestech Corporation) [32]. The results obtained using this device correlated well with measures obtained by other means [33]. The device was calibrated each day before its use. Total cholesterol (TC), high density lipoprotein (HDL), low density lipoprotein (LDL), triglycerides (TG) levels were determined. To define abnormal lipid/lipoprotein level, we used cut-off points suggested in American Heart Association guidelines: TC (≥200 mg/dL), HDL (</= 45 mg/dL), LDL (≥/=130 mg/dL), and TG (0–9 years of age ≥ 100 mg/dL; 10–19 years of age ≥ 130 mg/dL) [34].

### Statistical analysis

Results of the study were developed using descriptive statistics: number (n), %, $\overline{\mathrm{x}}$ (mean), Me – median, and standard deviation (SD). Both parametric and non-parametric tests were used to analyze the variables. The choice of the parametric test depended on the fulfilling its basic assumptions, i.e. the conformity of the distributions of the tested variables with the normal distribution, which were verified by the Shapiro-Wilk test. The student’s t-test was used for independent variables or, alternatively, non-parametric Mann-Whitney U test. The analysis of variables having the character of qualitative data was carried out with the Pearson chi-square test. The correlation of two variables with a normal distribution was determined with the Pearson’s linear correlation coefficient, and for the variables that did not meet the criterion of the normality of distribution, the Spearman rank correlation coefficient was calculated. Odds ratios were calculated with 95% confidence interval. Statistical power of the study was 0.88 (max. Error 12%). Statistical significance was established as a *p* value less than 0.05. Calculations were performed with Statistica 10.0.

## Results

The sample consisted of 69 participants (30 girls, 39 boys). In all, 14.5% of participants were overweight and 20.3% obese. Almost half of study group (47.8%) did not meet a recommendation of at least 60 min of MVPA daily. Descriptive characteristics of the study sample are shown in Table 1.

**Table 1:** Detailed characteristics of the group

Variable	Values
Age (years)	
6–12 ^a^	41 (59.4)
13–17 ^a^	28 (40.6)
Age ^b^	11.6 (2.74)
Body height ^b^	151.3 (14.25)
Body mass ^b^	48.2 (16.74)
BMI	20.4 (4.58)
Sex ^a^	
Girl	30 (43.5)
Boy	39 (56.5)
BMI percentile ^b^	63.4 (30.54)
Body mass category ^a^	
Underweight	2 (2.9)
Healthy weight	43 (62.3)
Overweight	10 (14.5)
Obesity	14 (20.3)
Body composition ^b^	
Fat [%]	22.1 (8.60)
FFM [%]	77.8 (8.59)
Muscle [%]	73.8 (8.09)
TBW [%]	57.0 (6.27)
Blood lipids ^b^	
TC [mg/dL]	161 (26.48)
HDL [mg/dL]	58 (14.68)
LDL [mg/dL]	90.12 (21.72)
TG [mg/dL]	86.6 (36.31)
Meet physical activity guidelines (based on daily MVPA) ^a^	
Yes (≥ 60 min)	36 (52.2)
No (< 60 min)	33 (47.8)

*BMI* body mass index, *FFM* free fat mass, *HDL* high density lipoprotein, *LDL* low density lipoprotein, *MVPA* moderate-to-vigorous physical activity, *TBW* total body water, *TC* total cholesterol, *TG* triglycerides

Data are expressed as: ^a^ – n (%); ^b^ – $\overline{\mathrm{x}}$ (SD)

Analysis of the association between results of physical activity and blood lipids showed a negative correlation between variables sedentary % and HDL, indicating that longer time spent on sedentary activities is associated with decreased HDL concentration (*p* = 0.046) (Table 2).

**Table 2:** Associations between physical activity and blood lipids

Physical activity	TC [mg/dl]	HDL [mg/dl]	LDL [mg/dl]	TG [mg/dl]
Sedentary [%]	*R* = − 0.17	*R* = − 0.24	*R* = − 0.09	*R* = − 0.00
	*p* = 0.162	*p* = 0.046	*p* = 0.543	*p* = 0.998
Light [%]	*R* = 0.19	*R* = 0.23	*R* = 0.12	*R* = 0.03
	*p* = 0.127	*p* = 0.054	*p* = 0.414	*p* = 0.832
Moderate [%]	*R* = 0.05	*R* = 0.17	*R* = − 0.07	*R* = 0.02
	*p* = 0.701	*p* = 0.164	*p* = 0.653	*p* = 0.859
Vigorous [%]	*R* = 0.11	*R* = − 0.04	*R* = 0.00	*R* = − 0.14
	*p* = 0.361	*p* = 0.708	*p* = 0.988	*p* = 0.260
Total MVPA [minutes]	R = 0.05	*R* = 0.15	*R* = 0.01	*R* = − 0.03
	*p* = 0.686	*p* = 0.233	*p* = 0.972	*p* = 0.838
MVPA [%]	*R* = 0.01	*R* = 0.12	*R* = − 0.05	*R* = − 0.06
	*p* = 0.930	*p* = 0.310	*p* = 0.731	*p* = 0.631
Average MVPA per day [minutes]	*R* = 0.02	*R* = 0.10	*R* = −0.01	R = − 0.04
	*p* = 0.901	*p* = 0.405	*p* = 0.962	*p* = 0.745
CPM	*R* = 0.07	*R* = 0.20	*R* = 0.05	*R* = − 0.06
	*p* = 0.556	*p* = 0.096	*p* = 0.716	*p* = 0.633
Steps per day	*R* = 0.05	*R* = 0.06	*R* = 0.05	*R* = 0.08
	*p* = 0.679	*p* = 0.575	*p* = 0.739	*p* = 0.504

*CPM* counts per minute, *HDL* high density lipoprotein, *LDL* low density lipoprotein, *MVPA* moderate-to-vigorous physical activity, *R* the result of the Spearman rank correlation test, *TC* total cholesterol, *TG* triglycerides

In Table 3, correlations between physical activity and body composition are presented. Participants who spent more time in the MVPA had a lower BMI percentile (*p* = 0.039).

**Table 3:** Associations between physical activity and body composition

Physical activity	Fat [%]	FFM [%]	Muscle [%]	TBW [%]	BMI percentile
% in Sedentary	*R* = −0.16	*R* = 0.01	*R* = 0.00	*R* = 0.16	*R* = 0.11
	*p* = 0.178	*p* = 0.955	*p* = 0.978	*p* = 0.181	*p* = 0.360
% in Light	*R* = −0.17	*R* = − 0.03	*R* = − 0.03	*R* = 0.17	*R* = 0.12
	*p* = 0.157	*p* = 0.794	*p* = 0.811	*p* = 0.155	*p* = 0.334
% in Moderate	*R* = −0.15	*R* = 0.06	*R* = 0.06	*R* = 0.15	*R* = 0.15
	*p* = 0.216	*p* = 0.627	*p* = 0.617	*p* = 0.221	*p* = 0.221
% in Vigorous	*R* = −0.18	*R* = 0.02	*R* = 0.02	*R* = 0.18	*R* = 0.03
	*p* = 0.120	*p* = 0.889	*p* = 0.867	*p* = 0.120	*p* = 0.797
Total MVPA	*R* = −0.19	*R* = 0.08	*R* = 0.08	*R* = 0.19	*R* = −0.24
	*p* = 0.108	*p* = 0.527	*p* = 0.504	*p* = 0.108	*p =* 0.039
% in MVPA	*R* = −0.19	*R* = 0.04	*R* = 0.04	*R* = 0.19	*R* = 0.05
	*p* = 0.109	*p* = 0.743	*p* = 0.721	*p* = 0.111	*p* = 0.701
Average MVPA per day	*R* = −0.19	*R* = 0.14	*R* = 0.14	*R* = 0.19	*R* = −0.23
	*p* = 0.118	*p* = 0.260	*p* = 0.247	*p* = 0.119	*p* = 0.054
CPM	*R* = −0.13	*R* = 0.08	*R* = 0.08	*R* = 0.13	*R* = −0.17
	*p* = 0.269	*p* = 0.506	*p* = 0.495	*p* = 0.261	*p* = 0.148
Steps Counts	*R* = −0.13	*R* = 0.07	*R* = 0.08	*R* = 0.14	*R* = −0.20
	*p* = 0.263	*p* = 0.554	*p* = 0.521	*p* = 0.252	*p* = 0.094

*BMI* body mass index, *CPM* counts per minute, *FFM* free fat mass, *MVPA* moderate-to-vigorous physical activity, *R* the result of the Spearman rank correlation test, *TBW* total body water

Table 4 presents the values of blood lipids/lipoproteins and body composition of participants meeting the recommendations for daily MVPA in relation to the participants of the study who did not meet these recommendations. A significant difference in body mass, free fat mass content (%) and muscle mass content (%) was noted among active and inactive participants; namely the active subjects had lower body mass (by 9.75 kg), higher free fat mass (by 5.51%) and higher muscle mass content (by 5.17%).

**Table 4:** Association between physical activity level with blood lipids and body composition

Variable	Meet physical activity guidelines						*p*
	Yes			No			
	$\overline{x}$	Me	SD	$\overline{x}$	Me	SD	
TC [mg/dL]	160.58	161.50	25.37	157.48	155.00	32.05	0.656
HDL [mg/dL]	60.06	57.00	15.01	56.45	55.00	16.89	0.194
LDL [mg/dL]	89.55	94.50	20.58	91.04	93.00	23.78	0.798
TG [mg/dL]	72.22	61.50	40.23	77.73	68.00	34.96	0.275
Body mass [kg]	43.57	42.80	15.01	53.32	53.40	17.52	0.015
Fat [%]	20.79	18.40	7.71	23.57	24.10	9.50	0.304
FFM [%]	80.51	82.35	7.30	75.00	74.91	9.19	0.007
Muscle [%]	76.27	78.08	6.89	71.10	71.16	8.66	0.008
TBW [%]	57.98	59.70	5.65	55.97	55.60	6.95	0.319
BMI percentile	19.51	18.75	4.10	21.48	20.60	4.99	0.079

*BMI* body mass index, *FFM* free fat mass, *HDL* high density lipoprotein, *LDL* low density lipoprotein, *TBW* total body water, *TC* total cholesterol, *TG* triglycerides

The cutoff for meeting the physical activity guidelines was at least 60 min daily of moderate-to-vigorous intensity physical activity

Table 5 presents odds ratios for the prevalence of abnormal values of blood lipids and fat content depending on the physical activity level. Inactive participants had more than 3 times higher risk for low HDL than subjects with a daily MVPA of at least 60 min (OR = 4.19). Inactive participants also had a more than 3 times higher risk for excessive body fat content and body weight (OR = 3.05 and 3.29, respectively).

**Table 5:** Odds ratios for the prevalence of abnormal values of blood lipids and fat content depending on the physical activity level

Meet physical activity guidelines				
Variable	Yes	No		*p*
	%	%	OR (95% CI)	
TC				
Acceptable	77.1	67.7	REF	
Abnormal	22.9	32.3	1.61 (0.54–4.78)	0.392
HDL				
Acceptable	88.9	65.6	REF	
Abnormal	11.1	34.4	4.19 (1,18-14,92)	0.027
LDL				
Acceptable	86.4	92.0	REF	
Abnormal	13.6	8.00	0.55 (0.08–3.64)	0.536
TG				
Acceptable	97.2	84.3	REF	
Abnormal	2.8	15.7	6.48 (0.71–8,79.4)	0.097
Fat [%]				
Healthy	80.6	57.6	REF	
Excessive	19.4	42.4	3.05 (1.04–8.95)	0.042
Body weight (based on BMI percentile)				
Healthy	77.8	51.5	REF	
Excessive	22.2	48.5	3.29 (1.16–9.33)	0.025

The cutoff for meeting the physical activity guidelines was at least 60 min daily of moderate-to-vigorous intensity physical activity

*BMI* body mass index, *HDL* high density lipoprotein, *LDL* low density lipoprotein, *REF* reference, *TC* total cholesterol, *TG* triglycerides

## Discussion

To our knowledge, this study is the first to compare the relationship between objectively measured physical activity levels (meeting versus not meeting daily MVPA recommendations) with blood lipid profile and body composition among a population of healthy children and adolescents. Presented issue is valuable due to the fact that increasing the energy expenditure, alongside a reduction in energy supply, seems to be the most important factor in weight reduction. Physical activity promotes proper energy balance, increase in muscle mass and development of new vascular capillaries. In addition, it improves vascular endothelial function, has anti-inflammatory effect and restores normal fibrinolytic activity [35]. It has been proven that short-time intense physical activity suppresses appetite, most likely by increasing body temperature, serum glucose and catecholamines, and production of endorphins [36]. In turn, long-term regular physical exercise is responsible for a steady gradual loss of fat mass. Post-exertion changes in the lipid profile may be associated with increased use of fatty acids, as energy substrates through skeletal muscle, reduction of hypertriglyceridemia and tissue resistance. The influence of physical activity on the increased activity of lipoprotein lipase, both in muscle and fat tissue, are also considered. Physical activity has a beneficial effect on glucose metabolism. Glucose uptake by skeletal muscles increases, and glycogenogenesis increases [37].

Physical activity influences irisin release, which already in a small concentration affects the “beigeing” of adipose tissue. Beige fat tissue (“beige” or “brite” beige-in-white) resembles brown adipose tissue (BAT) with structure and function [38]. Brown adipocytes are formed from myoblasts. They come from a common precursor line with skeletal muscle cells. Brown adipocytes, like white fat cells, function as an endocrine organ. They secrete adipokines to a lesser extent. They have a very high heat production capacity due to the high content of thermogenin in the mitochondrial inner membrane. Brown fat tissue positively affects the metabolism, prevents hypothermia, as well as obesity and related diseases. Brown adipose tissue in adults can be found mainly in the supraclavicular, perirenal, pericardial, inter-scapular and paraspinal region, especially in response to lowering of the environment temperature and beta-adrenergic stimulation. It influences the improvement of glucose tolerance significantly and the reduction of fasting insulin levels [39].

During physical activity skeletal muscles act as an endocrine organ secreting among others, myostatin. The main action of myostatin is inhibition of myoblasts proliferation. Both long-term and short-term physical exercise decrease myostatin concentration in muscle and blood serum. In addition, it has been proven that reduced myostatin concentration lowers fat content and improves glucose metabolism. As reported, increased muscle mass can be treated as an “anti-obesity buffer” [40].

Despite many health benefits associated with regular physical activity, the present study found a large percent of children and adolescents who did not meet recommended physical activity levels. This group was over 3 times more likely to have abnormal (low) values of HDL compared to the subjects meeting the recommendation. Moreover, participants not meeting the recommendation for physical activity were over 3 times more likely to have excessive body weight and fat content. We also demonstrated that participants meeting the minimum recommendations for daily MVPA in relation to the participants who did not meet these recommendations had higher free fat mass (by 5.51%) and higher muscle mass content (by 5.17%). There was no discernible association between daily MVPA and TC, LDL and TG.

According to Eisenmann correlations between physical activity and blood lipids in young populations are often low or nonsignificant, however, the data are inconsistent [41]. Armstrong et al. suggested that HDL is higher, and LDL and TG are lower in more active than inactive youth [42]. Katzmarzyk et al. also confirmed negative correlations between level of physical activity and TG and LDL, as well positive for HDL. The authors suggested that sex differences in the relationship between physical activity and coronary heart disease (CHD) had small risk factors, if they existed [11]. Similar results were obtained by Suter and Hawes, who examined children from 10 to 15 years old. In their analyses, physical activity level (from a 7-day recall) was the best predictor of HDL, TG, and LDL levels, while BMI, and dietary fat intake were not shown to be significant predictors. In boys, a high level of physical activity was related to higher concentrations of HDL, as well as to lower concentrations of LDL and TG. In girls, physical activity was positively related only to HDL [43]. Results of another study that examined the relationship between MVPA and dyslipidemia in youth, observed a relationship between high-risk HDL cholesterol and TG values and no clear relationship between MVPA and high-risk LDL cholesterol values [19]. These results are similar to results obtained in our study, in which we also demonstrate that participants with daily MVPA less than 60 min had higher odds for high-risk HDL cholesterol (OR = 4.19). Although in our study there was no association between physical activity levels and TG.

Results contrary to ours were obtained by Schmidt et al. who found no difference between active and non-active children and HDL. Differences were shown between the two groups in TC, LDL, TG, and body fat percentage. This may suggest that coronary risk factors may be reduced in children with higher activity levels [44]. Raitakari et al. performed a cross-sectional study among young Finnish people from 9 to 24 years of age. Participants were divided into tertiles based on their level of self-reported MVPA. The results show that a high level of physical activity was associated with high HDL concentrations, and low levels of TG in boys. However, in girls, the influence of physical activity was evident only on TG level. In both genders, physical activity was inversely associated with obesity [45]. DuRant et al. examined the relationships among indicators of physical activity, physical fitness, and body composition with serum lipid and lipoproteins levels in children aged 3–5 years. According to authors, physical activity appeared to have an indirect association with serum lipid and lipoprotein values through its relationship with higher fitness levels and lower levels of body fat [46].

Inconsistent findings reported on associations between physical activity levels and lipids and lipoproteins may be the result of errors inherent in the methods used to measure physical activity; different non-objective tools for measuring physical activity levels and various cut-off points in determining physical activity levels are used. This is well illustrated by the results of Hearst et al. in which three different methods to assess physical activity were applied to one group of adolescents. The self-administered survey DPAR3 showed that the MVPA recommendation of WHO was achieved by 69% of respondents, 36% by a MAQ questionnaire, and results from an accelerator indicated only 6% [47]. Self-report questionnaires for assessment of physical activity have the potential for measurement error that can lead to incorrect conclusions about physical activity behaviors and can bias study results [48]. In addition, self-report questionnaires recall methods that are common in physical activity research with youth and adults that may not be appropriate for young children because of their inability to accurately recall their own activity levels [49]. Therefore, objective methods such as accelerometers used in our study are the most feasible measures for young children.

Intervention studies have also demonstrated improvements in lipid-lipoprotein profiles with increases in physical activity in youth. For example, Chen et al. indicated that in overweight children, after a short-term (2 week) rigorous diet and exercise regimen TC, LDL, TG and fat content were reduced, with no change in HDL observed [50]. In addition, Tolfrey et al. observed that over an intervention period (12 weeks of training involved stationary cycling for 30 min, 3 times a week), LDL decreased and HDL increased [51]. Janssen and LeBlanc in their meta-analysis examined the effect of exercise interventions on changes in blood lipids and lipoproteins in children and adolescents. The authors concluded that due to the different designs of the interventions, the nature of the dose-response relation between exercise and blood lipids/lipoproteins in the pediatric population remains unclear. However, they concluded that to achieve substantive health benefits, physical activity should be of at least a moderate intensity, and it should be recognized that vigorous intensity activities may provide an even greater benefit [52]. Therefore, promotion of physical activity is important, especially among the most sedentary children for preventing obesity and premature cardiovascular disease [53].

The results of this study clearly show that meeting physical activity guidelines is related to higher free fat mass and muscle content. Although some cross-sectional studies suggest that obese children are less active than non-obese children, a relationship between physical activity and adiposity in the general pediatric population is not clearly established [41]. A meta-analysis suggests there is a small to moderate relationship between physical activity and body fat in children and adolescents, but the extent of the relationship depends on the method of physical activity assessment. The authors recommended direct measurements of physical activity in assessments of the relationship of activity levels with health [54].

In summary, the present study found that meeting the WHO recommendation for physical activity had a positive association with both HDL level and free fat mass in a pediatric population. Health promotion programs should focus on strategies that help children and adolescents meet the current guidelines of at least 60 min per day of MVPA to prevent serious health problems later in their lives.

### Strengths and limitations

This study has some strong points. We have differentiated intensity of participant physical activity (*meeting* versus *not meeting WHO recommendations for daily MVPA*) utilizing objective measures. Accelerometers provide a reliable and objective measure of physical activity in pediatric populations. A limitation of previous observational studies is that data regarding the level and intensity of physical activity was often obtained through subjective, self-report questionnaires. Incorrect estimations of physical activity may have masked the true association between physical activity and lipid/lipoprotein levels in the past.

There are also a number of potential limitations of the study that need to be taken into account when interpreting the results. The primary study limitation was that it was observational and cross-sectional in nature, limiting the ability to make causal inferences about the association between MVPA and lipid/lipoprotein levels. Another limitation of the study was the relatively small sample size. Furthermore, the age range of the participants (6–17 years) should be considered as a limitation due to differences in participant maturation, which has an important impact on levels of lipids and lipoproteins [55]. Despite a suspected confounding effect of hormonal changes occurring during puberty, the present study was able to document an association of physical activity and blood lipid/lipoprotein levels in the participants studied. Future studies with more potential factors and larger participant groups are needed to confirm our results.

## Conclusion

Spending at least 60 min daily on MVPA may be associated with a more favorable HDL and body fat level in children and adolescents. The association between insufficient daily MVPA with high-risk HDL and body fat percentage offers encouraging information for clinicians and other health care practitioners who are concerned about promoting physical activity in young patients. It is essential to design more effective interventions to stimulate physical activity at every stage of life, or to counteract the growing trend towards inactivity.

## Abbreviations

BIA: Bioelectrical impedance analysis
BMI: Body mass index
CPM: Counts per minute
FFM: Free fat mass
HDL: High density lipoprotein
LDL: Low density lipoprotein
MVPA: Moderate-to-vigorous intensity physical activity
TBW: Total body water
TC: Total cholesterol
TG: Triglycerides
WHO: World Health Organization
